# Long‐term impact of the COVID‐19 pandemic on facility‐ and home‐dwelling people with dementia: Perspectives from professionals involved in dementia care

**DOI:** 10.1111/ggi.14465

**Published:** 2022-09-06

**Authors:** Kana Kazawa, Tatsuhiko Kubo, Masahiro Akishita, Shinya Ishii

**Affiliations:** ^1^ Department of Medicine for Integrated Approach to Social Inclusion, Graduate School of Biomedical and Health Sciences Hiroshima University Hiroshima Japan; ^2^ Department of Public Health and Health Policy, Graduate School of Biomedical and Health Sciences Hiroshima University Hiroshima Japan; ^3^ Department of Geriatric Medicine, Graduate School of Medicine The University of Tokyo Tokyo Japan

**Keywords:** community, COVID‐19, dementia, long‐term care

## Abstract

**Aim:**

The present study aimed to investigate the impact of the coronavirus disease 2019 (COVID‐19) pandemic on facility‐ and home‐dwelling people with dementia (PWD).

**Methods:**

This observational study included two anonymous online survey questionnaires to explore the impact of the first wave of the COVID‐19 pandemic in Japan and the long‐term impact during the 2 years from the onset of the pandemic. The participants were medical and long‐term care facilities representatives for older people (945 facilities in the first survey, 686 in the second), and care managers (751 in the first survey, 241 in the second). A χ^2^‐test was carried out between the two surveys.

**Results:**

For facility‐dwelling PWD, activities that stimulate cognitive and physical functioning increased significantly compared with the first wave of the pandemic (*P* < 0.05). Also, a decline in cognitive and walking functions and falls increased in the second survey compared with the first (*P* < 0.01). For home‐dwelling PWD, the broader impact of the pandemic on support for activities of daily living, social interaction and provision of medical care did not mitigate. The high prevalence of cognitive and physical functional decline in the first survey was similar in the second.

**Conclusions:**

The prolonged COVID‐19 pandemic produced changes in the lives of home‐ and facility‐dwelling PWD, with widespread negative consequences for them. Our findings are useful to consider preventive supports to mitigate or avoid functional decline and symptom exacerbation in PWD due to changes in their living environment and the care they receive in the COVID‐19 era. **Geriatr Gerontol Int 2022; 22: 832–838**.

## Introduction

The coronavirus disease 2019 (COVID‐19) pandemic has severely affected the delivery of face‐to‐face dementia care in facilities and at home. For home‐dwelling people with dementia (PWD), social isolation, reduced informal community activities and restrictions on access to paid service have led to a lack of cognitive and physical stimulation in their daily lives, resulting in a decline in cognitive and physical functions, and worsening of behavioral and psychological symptoms of dementia (BPSD).^1^, ^2^, ^3^, ^4^ For facility‐dwelling PWD, emotional changes, such as loneliness and anger, occur due to restrictions on family visits.^5^ Also, families provide necessary information on the values and lifestyle of PWD to enable the facility staff to understand the residents better and implement individualized dementia care.^6^ However, during the COVID‐19 pandemic, communication difficulties between family members and facility staff create a gap between desired care and actual care of PWD, leading to deterioration of cognitive and physical functions of the residents.^7^

In Japan, older people aged ≥65 years who require daily long‐term care account for 18.3% of the total older population.^8^ Approximately 55.0% of this population is reported to have dementia.^9^ In addition, a survey report on 35 700 people with early‐onset dementia showed that 70% of the respondents were certified as people who require care under the long‐term care insurance system.^10^ Since the spring of 2020, the Japanese government has repeatedly announced a policy of mild lockdown and “stay home” directives to prevent the spread of COVID‐19, and long‐term care facilities have implemented these intermittent visitation restrictions.^11^ Thus, social interaction activities in the community have been reduced, and public long‐term care insurance services, especially short‐stay and day‐care services, have been temporarily reduced or suspended.^12^ With the prolongation of the COVID‐19 pandemic, the changes in lifestyle and the unfavorable impact of the pandemic on the health statuses of PWD, associated with the aforementioned public health and individual infection control measures, need to be carefully evaluated. Unfortunately, the end of the COVID‐19 pandemic is not yet in sight. Understanding how PWD have been affected for the approximately 2‐year duration of the pandemic provides a valuable basis for implementing effective infection prevention while minimizing unfavorable impacts in the future. Therefore, the present study aimed to assess the impact of the approximately 2‐year COVID‐19 pandemic on PWD. The key research questions were:

1. How were the lives of facility‐ and home‐dwelling PWD affected?

2. How were the physical and psychosocial conditions of facility‐ and home‐dwelling PWD affected?

## Methods

### Study design

This was a repeated cross‐sectional study carried out by administering an online survey on the short‐term impact of the spread of COVID‐19 (the first survey: June to July 2020) and the long‐term (approximately 2 years) impact of the pandemic (the second survey: October to December 2021) in Japan. The present study is a collaborative qualitative study involving Hiroshima University and the Japan Geriatrics Society.

### Participants and recruitment

For the survey of facility‐dwelling PWD, the participants were representatives of medical and long‐term care facilities for older people throughout Japan registered with the following organizations: Japan Association of Medical and Care Facilities, Japan Psychiatric Hospitals Association, Japan Association of Geriatric Health Services Facilities, Japanese Council of Senior Citizens Welfare Service, Japan Group‐Home Association for People with Dementia and Japanese Council of Daily Life Long‐Term Care Service Facilities. Medical facilities included hospitals that specialize in the treatment and recuperation of PWD, mental illness and chronic diseases that require long‐term care. Long‐term care facilities included care homes, group homes for PWD and other facilities that support those who need daily long‐term care.

For the survey of home‐dwelling PWD, the participants were care managers registered with the Japan Care Manager Association who planned and coordinated public long‐term care insurance services of PWD.

The participants were provided with an online document requesting them to take part in the study and a URL link to the survey questionnaire through their organization. Representatives of the medical and long‐term care facilities were allowed to respond only once per facility. Google Forms (Googleplex, Mountain View, CA, USA) was used for the survey.

### Data collection and analysis

In the present study, we carried out an online anonymized and self‐administered questionnaire, because we aimed to obtain large‐scale data in a timely manner without running the risk of spreading infection or placing burden on targeted facilities. We also considered that this survey method would enable the respondents to answer openly difficulties in care provision and service accessibility issues for care users. The questionnaire consisted of questions regarding the attributes of the participants and the impact of the COVID‐19 pandemic on the lives of PWD and their families, including their physical and psychosocial conditions (SuppInfo 1 Text).

A descriptive statistic was carried out and a χ^2^‐test was performed between the data from the first survey and the data from the second survey. The severity of dementia was classified based on the “Criteria for determination of the daily life independence level of the elderly with dementia” defined by the Ministry of Health, Labor and Welfare in Japan. There are six levels in the original criteria: independence, grades I–IV, and M. In the present study, severe dementia was defined as the original grade III level or higher (symptoms, behavior or difficulty in communication that interfere with the person's daily life are observed frequently and require care). Mild‐to‐moderate dementia was defined as grade I–II.

The spss software (version 27.0; manufactured by IBM, Armonk, NY, USA) was used for analysis, and the significance level was set at 5%.

### Ethical considerations

The participants were provided with a web‐based explanation of the purpose, content and privacy of the study. The participants responded to the questionnaire anonymously and were deemed to have provided consent to participate in the study on submission of the online survey form. Therefore, approval from an ethics committee was not necessary. The present study was carried out by Hiroshima University in collaboration with the COVID‐19 response team of the Japan Geriatrics Society and it conforms to the provisions of the Declaration of Helsinki.

## Results

In the first and second surveys, 945 and 686 representatives of medical and long‐term care facilities and 751 and 241 care managers, respectively, responded. All responses were analyzed (SuppInfo 2 Table). The first survey was carried out immediately after the initial wave and did not set up a question about cluster infections; however, 16 facilities responded in the second survey that they had experienced it.

### Impact of the COVID‐19 pandemic on the lives of PWD

**Figure 1:**
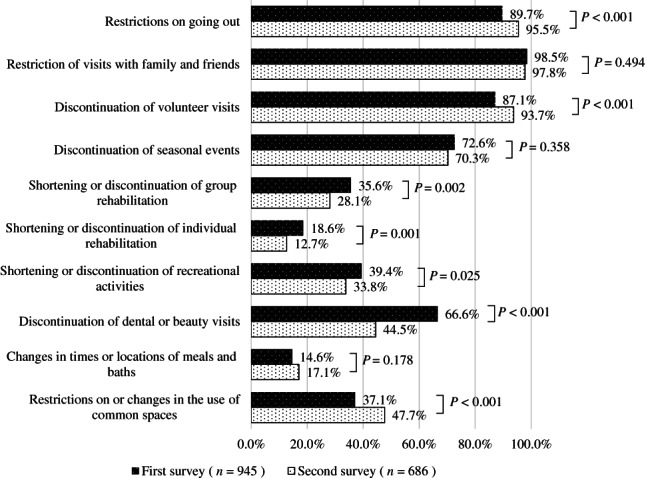
The impact of the coronavirus disease 2019 pandemic on facility‐dwelling people with dementia.

Figure 1 shows the impact on the lives of facility‐dwelling PWD. The percentage of respondents who indicated that they implemented measures to prevent infection, such as limiting outings and interactions with non‐family members, and changing the use of common spaces, was significantly higher in the second survey than in the first survey (*P* < 0.001). However, the percentage of respondents in the second survey who indicated that they were shortening or discontinuing group rehabilitation, individual rehabilitation and recreational activities, which are necessary for maintaining the functions and interactions of the residents, decreased by approximately 10–20% compared with the first survey (*P* < 0.05). Restriction of visits with family and friends continued at a high rate in the second survey.

**Figure 2:**
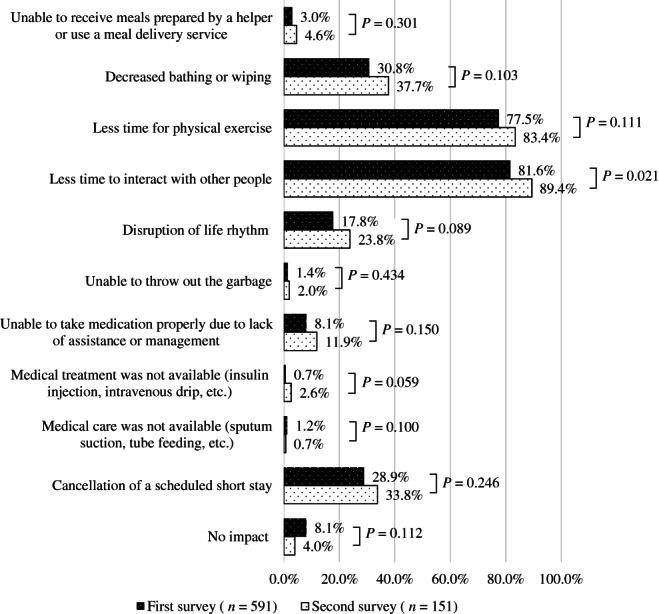
The impact of the coronavirus disease 2019 pandemic on home‐dwelling people with dementia.

Regarding the impact on the lives of home‐dwelling PWD (Fig. 2), we asked those who responded that the use of public long‐term care insurance services for PWD had been affected (591 [78.7%] in the first survey; 151 [62.7%] in the second survey) about the impact of these changes on the lives of PWD. The percentage of respondents in the second survey who answered that the time spent on physical exercise and interaction with other people had decreased was slightly increased compared with the first survey (*P* = 0.111, 0.021, respectively). We observed that the number of respondents in the second survey who said PWD were unable to receive support for activities of daily living (ADL), such as eating, bathing and wiping, had a slight increasing trend compared with the first survey. The percentage of PWD who required medical treatment or care, but were unable to receive it in the first survey, was not changed in the second survey.

### Impact of the pandemic on the conditions of PWD and their families

**Figure 3:**
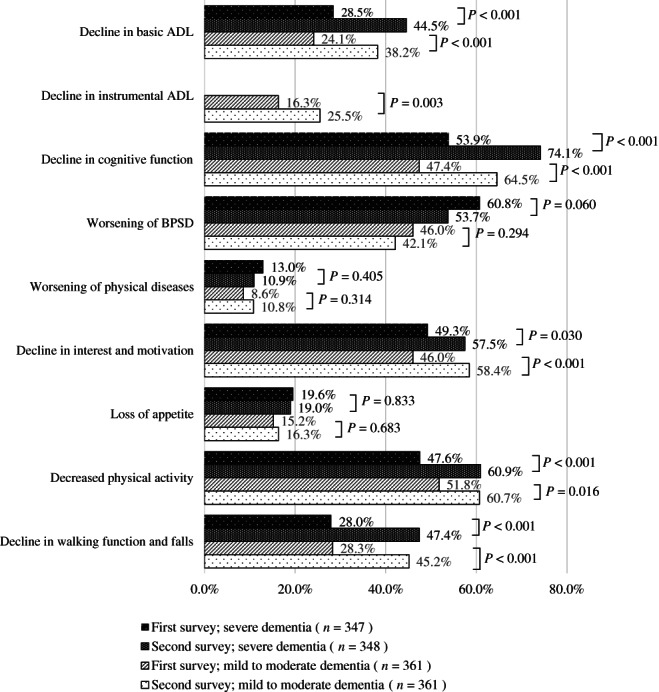
Changes in the conditions of facility‐dwelling people with dementia during the coronavirus disease 2019 pandemic. ADL, activities of daily living; BPSD, behavioral and psychological symptoms of dementia.

**Figure 4:**
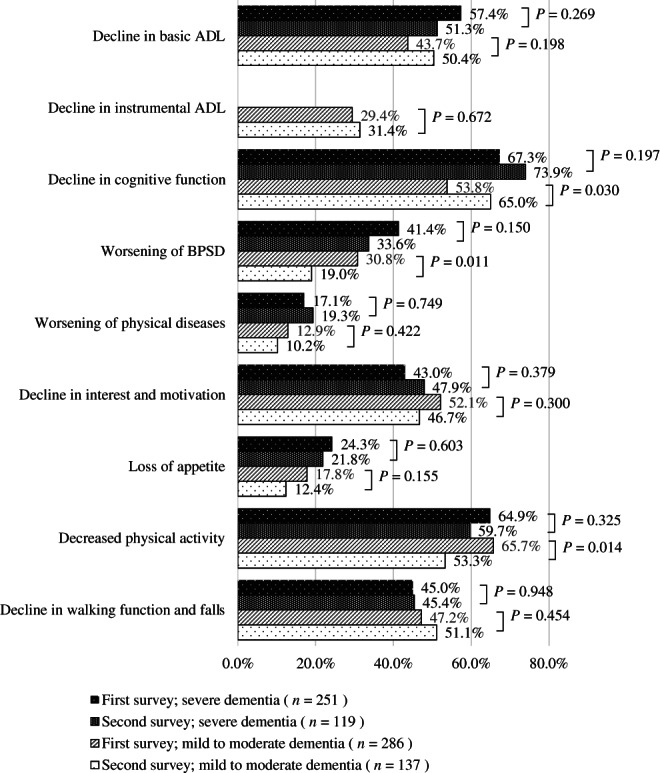
Changes in the conditions of home‐dwelling people with dementia coronavirus disease 2019 pandemic. ADL, activities of daily living; BPSD, behavioral and psychological symptoms of dementia.

Figures 3 and 4 show the changes in the conditions of PWD. In the first survey, 361 facilities (38.5%) and 286 care managers (38.1%), and in the second survey, 361 facilities (52.6%) and 137 care managers (56.8%) responded that the pandemic had an unfavorable impact on PWD.

Regarding facility‐dwelling PWD, reports of decline in ADL/instrumental ADL, cognitive function, interest and motivation, physical activity, and walking function/falls in the second survey increased significantly compared with the first survey (*P* < 0.05).

Regarding home‐dwelling PWD, the high prevalence of cognitive and physical functional decline in the first survey was similar in the second, whereas appearance or worsening of BPSD, appetite and physical activity showed a decreasing trend in the second survey compared with the first. Regarding the severity of dementia, a decline in basic ADL decreased in people with severe dementia (57.4% in the first survey, 51.3% in the second), whereas it increased in those with mild‐to‐moderate dementia (43.7% in the first survey, 50.4% in the second), although these differences were not statistically significant.

**Figure 5:**
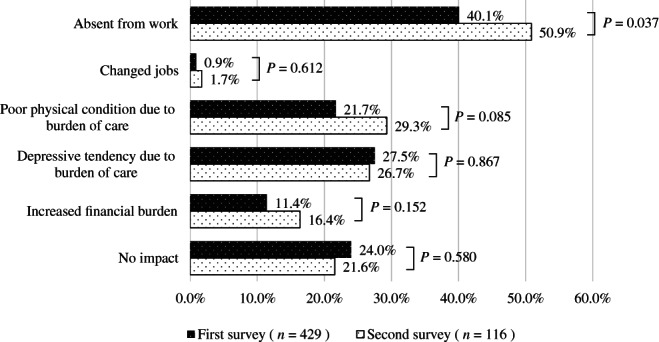
The impact of the coronavirus disease 2019 pandemic on the families of home‐dwelling people with dementia.

Figure 5 shows the impact on the families of home‐dwelling PWD. A total of 429 (57.1%) respondents in the first survey and 116 (48.1%) in the second survey reported that family members took care of PWD temporarily due to the reduction or interruption of public long‐term care insurance services. In both surveys, respondents indicated that almost half of family members took a leave of absence from work owing to the burden of care, and the percentage of them in the second survey increased compared with the first survey (40.1% in the first survey and 50.9% in the second, *P* = 0.037). In the second survey, the number of more respondents indicated that family members changed their jobs, had health problems due to the burden of care and experienced further financial problems increased slightly.

## Discussion

We investigated the impact of the COVID‐19 pandemic on PWD. The main finding of this study was that there is a difference between the impact of the pandemic on the lives of PWD living in facilities and those living at home. For facility‐dwelling PWD, activities that stimulate cognitive and physical functioning increased after approximately 2 years compared with the period of the first wave of the pandemic, whereas the broader impact of the pandemic on the lives of home‐dwelling PWD showed no trend toward alleviation. This difference might be related to the fact that, in care facilities, formal caregivers are more likely to take the initiative in separating living spaces and encouraging activities based on social distancing and infection control measures. Facilities also have easier access to state and private information resources on exercise and cognitive training to mitigate the negative effects of the pandemic on PWD.^13^, ^14^

In contrast, family caregivers of home‐dwelling PWD might be conflicted regarding the advantages of using long‐term care insurance services and social interaction, such as maintaining the functions of PWD and reducing the burden of care, and the disadvantages of the risk of infection during the COVID‐19 pandemic.^15^ During the difficult decision‐making process of considering the use of long‐term care insurance services by families of PWD, detailed explanations of infection control measures by healthcare providers and discussions about care priority have been shown to reduce caregiver conflicts and lead to cooperative, person‐centered dementia care.^16^ These results suggest the necessity of these practices among PWD, family caregivers and healthcare providers as appropriate during repeated infection epidemics. Furthermore, in the present study, most facilities have implemented safety measures, such as restricting the residents from leaving the facilities and interacting with outsiders, including families, to protect their lives and reduce the risk of infection.

As shown in many previous reports, the communication‐aid tools, such as telephone, e‐mail or videoconferencing, are useful for communication between PWD and their families to promote their mental health,^5^, ^17^ and for evaluating care and discussing care priorities and plans between facility staff and families.^6^ It is expected that these communication tools will be actively used according to the preferences and cognitive functions of PWD.

Regarding the impact of the COVID‐19 pandemic on the conditions of PWD and their families, the present study results showed that facility‐ and home‐dwelling PWD were extensively affected, and that the prevalence of cognitive functional decline was high. In the present study, few facilities reported that they had experienced COVID‐19 cluster infections. Therefore, the results for facility‐dwelling PWD were not caused by cluster infections, but by long‐term infection control measures.

The decreased cognitive function, physical activity, social interaction, and interest and motivation might have affected the physical and psychosocial conditions of PWD and their families, causing a vicious cycle. Furthermore, it should be noted that the percentage of home‐dwelling PWD with mild‐to‐moderate dementia who showed a decline in ADL increased from the first to the second survey. The decline in their cognitive function due to long‐term infection control measures leads to a decline in ADL, which might prevent them from maintaining their own lifestyles, leading to further deterioration in cognitive and physical functions, and the appearance and worsening of BPSD.^2^, ^18^ In addition, the decline in ADL might lead to an increased risk of COVID‐19 infection due to worsening nutritional status, physical function and underlying health conditions.^19^, ^20^ Providing information and care resources, such as indoor exercise practice guides for older people^13^ and online activities,^14^ according to their preferences, conditions and lifestyle of PWD would be useful in mitigating the negative effects of the pandemic.^21^, ^22^

Regarding the impact on the families of home‐dwelling PWD, the reduction or interruption of public long‐term care insurance services has increased their burden. Caregiver burden is a predictor of the institutionalization of PWD,^23^ it has been reported that the use of home visit services and short stays can support the daily lives and well‐being of the caregivers.^24^ In the future, it will be necessary to develop a system that allows multiple care facilities to collaborate for the provision of sustainable services, direct interaction support to avoid social isolation of families, and information sharing among residents, government and medical institutions to enable timely intervention for families in need of support.^25^

The present study had limitations. First, this was focused on formal caregivers, and involved the broad examination of the effects of the COVID‐19 pandemic on PWD; thus, the impact of individual dementia characteristics at the individual level were not analyzed. In addition, PWD who were not registered for medical and long‐term care insurance services were not followed. Therefore, further research needs to address the selection bias and provide implications for specific support strategies. Second, as the two surveys were anonymous and independently conducted, we were not able to evaluate within‐facility longitudinal changes. Therefore, these results must be interpreted with caution. Nevertheless, this is the first long‐term and comprehensive survey that assessed the physical and psychosocial conditions of PWD under the COVID‐19 pandemic.

The present study determined the long‐term impact of the COVID‐19 pandemic on facility‐ and home‐dwelling PWD using an online survey. This survey showed the impact of the COVID‐19 pandemic on PWD living in facilities and at home, and that PWD have experienced a wide range of adverse effects, including limitation of daily activities, reduced social interaction, and decline in cognitive and physical function. Compared with the first wave of the COVID‐19 pandemic, facility‐dwelling PWD experienced a long‐term increase in the activities that stimulate cognitive and physical functions and interactions, whereas home‐dwelling PWD experienced a decrease in these activities.

Regarding dementia care during the COVID‐19 pandemic, it is essential for formal caregivers to share information on the risk of infection carefully, unfavorable effects associated with infection control measures, and care priorities with PWD and their families, and assess their accessibility to information and care resources, and help them secure resources when necessary.

## Disclosure statement

The authors declare no conflict of interest.

## Supporting information


**Appendix S1.** The questionnaire used for the study
ADL, activities of daily living; BPSD, behavioral and psychological symptoms of dementia; PWD, people with dementia.

**Appendix S2.** Characteristics of the participants

## Data Availability

The datasets analyzed in the present study are not publicly available. This study was conducted by Hiroshima University in collaboration with the COVID‐19 response team of the Japan Geriatrics Society. Informed consent for the secondary use of the data was not obtained from the participants.
