# Supplementary material for: Long‐term impact of the COVID‐19 pandemic on facility‐ and home‐dwelling people with dementia: Perspectives from professionals involved in dementia care
Source: Geriatr Gerontol Int. 2022 Sep 6;22(10):832–8. doi: 10.1111/ggi.14465 (PMC9538434; doi:10.1111/ggi.14465)
Supplement: Supplementary file 1 — Appendix S1. The questionnaire used for the study ADL, activities of daily living; BPSD, behavioral and psychological symptoms of dementia; PWD, people with dementia. [file GGI-22-832-s002.pdf]

SuppInfo 1 Text. Questionnaire used in the present study

The questions relevant to this study were extracted and are presented below.

1. Attributes

- Type of facility
- Location of the facility

2. Impact of the pandemic on the lives of people with dementia (PWD)

1) Questions for medical and long-term care facilities

- Has your facility experienced COVID-19 cluster infections? (The second survey only)
- Please indicate the measures you have taken to prevent infection.

□ Restrictions on going out	☐ Restriction of visits with family and friends
□ Discontinuation of volunteer visits	☐ Discontinuation of seasonal events
□ Shortening or discontinuation of group rehabilitation	☐ Shortening or discontinuation of individual rehabilitation
□ Shortening or discontinuation of recreational activities	☐ Discontinuation of dental or beauty visits
□ Changes in times or locations of meals and baths	☐ Restrictions on or changes in the use of common spaces

2) Questions for care managers

- During the COVID-19 pandemic, did any of the PWD in your charge show any changes in the use of long-term care insurance service?

□ Yes □ No

- How did the changes in the utilization of long-term care insurance service affect the lives of the PWD you were in charge of? Please select all that apply.

□ Unable to receive meals prepared by a helper or use a meal delivery service
□ Decreased bathing or wiping
□ Less time to interact with other people
☐ Less time for physical exercise
□ Unable to throw out the garbage
☐ Disruption of life rhythm
□ Unable to take medication properly due to lack of assistance or management
□ Medical treatment was not available (insulin injection, intravenous drip, etc.)
□ Medical care was not available (sputum suction, tube feeding, etc.)
□ Cancellation of a scheduled short stay
□ No impact

SuppInfo 1 Text. Continued.

3. Impact of the pandemic on conditions of PWD

- Please select all that apply regarding the impact of the restriction of daily activities due to infection control measures on the conditions of PWD.

□ Decline in basic ADL	☐ Decline in cognitive function
□ Worsening of BPSD	☐ Worsening of physical diseases
□ Decline in interest and motivation	☐ Loss of appetite
□ Decreased physical activity.	☐ Decline in walking function and falls

ADL, activities of daily living; BPSD, behavioral and psychological symptoms of dementia

- Please select all that apply regarding the impact of the restriction of daily activities due to infection control measures on the condition of persons with mild to moderate dementia.

□ Decline in basic ADL	☐ Decline in instrumental ADL
□ Decline in cognitive function	☐ Worsening of BPSD
□ Worsening of physical diseases	☐ Decline in interest and motivation
□ Loss of appetite	☐ Decreased physical activity
□ Decline in walking and falls	

4. Impact of the pandemic on families of home-dwelling PWD (questions for care managers).

- Has the change in service utilization by PWD during the COVID-19 pandemic increased the amount of care provided by their families?

□ Yes □ No □ Not sure

5. What impact did the increased amount of care for PWD have on their families? Please select all that apply.

□ Absences from work
□ Poor physical condition due to burden of care
□ Increased financial burden
□ No impact
