# Supplementary material for: Long‐term impact of the COVID‐19 pandemic on facility‐ and home‐dwelling people with dementia: Perspectives from professionals involved in dementia care
Source: Geriatr Gerontol Int. 2022 Sep 6;22(10):832–8. doi: 10.1111/ggi.14465 (PMC9538434; doi:10.1111/ggi.14465)
Supplement: Supplementary file 2 — Appendix S2. Characteristics of the participants [file GGI-22-832-s001.docx]

SuppInfo 2 Table. Characteristics of the participants

| Attribute | | | First  survey | | Second survey | | p-value |
| --- | --- | --- | --- | --- | --- | --- | --- |
|  |  |  | n | % | n | % |  |
| Medical and long-term care facilities | | | 945 |  | 686 |  |  |
|  | Types of facility | |  |  |  |  |  |
|  | Medical facilities | | 141 | 14.9 | 14 | 2.0 |  |
|  |  | Dementia treatment unit | 43 | 4.6 | 3 | 0.4 | .757 |
|  |  | Medical recuperation unit | 50 | 5.3 | 6 | 0.9 |  |
|  |  | Others | 48 | 5.1 | 5 | 0.7 |  |
|  | Long-term care facilities | | 804 | 85.1 | 672 | 98.0 |  |
|  |  | Intensive care home for older people | 257 | 27.2 | 156 | 22.7 | <.001 |
|  |  | Group home for older people with dementia | 228 | 24.1 | 371 | 54.1 |  |
|  |  | Others | 319 | 33.8 | 145 | 21.1 |  |
|  | Location of the facility | |  |  |  |  |  |
|  |  | Prefectures under state of emergency between April and May 2020^†^ | 254 | 26.9 |  |  |  |
|  |  | Prefectures under specific precautions between April and May 2020^‡^ | 169 | 17.9 |  |  |  |
|  |  | Prefectures within the top 25% of places with the highest cumulative number of new positive COVID-19 cases per population between January 2020 and November 2021^§^ |  |  | 251 | 36.6 |  |
|  |  | Prefectures within the top 25% to 50% of places with the highest cumulative number of new positive COVID-19 cases per population between January 2020 and November 2021^§^ |  |  | 164 | 23.9 |  |
|  |  | Others (including no response) | 522 | 55.2 | 271 | 39.5 |  |
| Care managers | | | 751 |  | 241 |  |  |
|  | Type of facility | |  |  |  |  |  |
|  |  | Home care support office | 691 | 92.0 | 221 | 91.7 | .953 |
|  |  | Community general support center | 37 | 4.9 | 13 | 5.4 |  |
|  |  | Others | 23 | 3.1 | 7 | 2.9 |  |

SuppInfo 2 Table. Continued.

| Attribute | | | First  survey | | Second survey | |  |
| --- | --- | --- | --- | --- | --- | --- | --- |
|  |  |  | n | % | n | % |  |
| Care managers | | | 751 |  | 241 |  |  |
|  | Location of the facility | |  |  |  |  |  |
|  |  | Prefectures under state of emergency between April and May 2020^†^ | 148 | 19.7 |  |  |  |
|  |  | Prefectures under specific precautions between April and May 2020^‡^ | 85 | 11.3 |  |  |  |
|  |  | Prefectures within the top 25% of places with the highest cumulative number of new positive COVID-19 cases per population between January 2020 and November 2021^§^ |  |  | 74 | 30.7 |  |
|  |  | Prefectures within the top 25% to 50% of places with the highest cumulative number of new positive COVID-19 cases per population between January 2020 and November 2021^§^ |  |  | 126 | 52.3 |  |
|  |  | Others (including no response) | 518 | 69.0 | 41 | 17.0 |  |
| ^†^ Seven prefectures where the spread of infection was most serious and the Japanese government had declared the state of emergency as of the time of the first survey (Tokyo, Kanagawa, Saitama, Chiba, Osaka, Hyogo, and Fukuoka). | | | | | | | |
| ^‡^ Six prefectures where the spread of infection was serious and the Japanese government had declared the specific precautions as of time of the first survey (Hokkaido, Ibaraki, Ishikawa, Gifu, Aichi, and Kyoto). | | | | | | | |
| ^§^ At the time of the second survey (November 1, 2021), the cumulative number of new positive cases was calculated for each prefecture. Thereafter, the cumulative number of new positive cases per 100,000 population (the population was calculated using the population estimate as of October 1, 2021, based on the national statistics survey) was calculated. The prefectures were then listed in order of the number of cumulative new positive cases, and the quartiles were used to classify the prefectures as follows: | | | | | | | |
|  | - Twelve prefectures within the top 25% (75th percentile or higher): Okinawa, Tokyo, Osaka, Kanagawa, Chiba, Saitama, Fukuoka, Hyogo, Aichi, Kyoto, Nara, and Hokkaido. | | | | | | |
|  | - Twelve prefectures within the top 25% to 50% ( the 50th to the 75th percentile): Gifu, Shiga, Gunma, Ibaraki, Mie, Kumamoto, Okayama, Tochigi, Hiroshima, Shizuoka, Oita, and Saga. | | | | | | |
|  | - Others. | |  |  |  |  |  |
